# Gap variability upon packing in organic photovoltaics

**DOI:** 10.1371/journal.pone.0234115

**Published:** 2020-06-16

**Authors:** D. López-Durán, Etienne Plésiat, Michal Krompiec, Emilio Artacho

**Affiliations:** 1 CIC Nanogune BRTA, San Sebastián, Spain; 2 Departamento de Química, Módulo 13, Universidad Autónoma de Madrid, Madrid, Spain; 3 Merck Chemicals Ltd., Chilworth Technical Centre, University Parkway, Southampton, United Kingdom; 4 School of Chemistry, University of Southampton, Highfield, Southampton, United Kingdom; 5 Theory of Condensed Matter, Cavendish Laboratory, University of Cambridge, Cambridge, United Kingdom; 6 Donostia International Physics Center DIPC, San Sebastián, Spain; 7 Ikerbasque, Basque Foundation for Science, Bilbao, Spain; San Jose State University, UNITED STATES

## Abstract

The variation of the HOMO-LUMO band gap is explored for varying packing arrangements of the 4*mod* BT-4TIC donor-acceptor molecule pair, by means of a high-throughput ab-initio random structure search of packing possibilities. 350 arrangements of the dimer have been relaxed from initial random dispositions, using non-local density-functional theory. We find that the electronic band gap varies within 0.3 eV, and that this magnitude, the binding energy, and the geometry are not significantly correlated. A clearly favoured structure is found with a binding energy of 1.75±0.07 eV, with all but three other arrangements displaying values of less than one third of this highest binding one, which involves the aliphatic chain of 4TIC.

## Introduction

The need to find easily renewable and environmentally friendly energy sources alternative to the traditional fossil fuels is nowadays a global quest. The solar energy is a promising candidate and solution-processed organic solar cells (OSCs) have attracted attention because of their low cost, light weight, mechanical flexibility, and potential application in large-area devices [1–3]. A typical OSC is formed by mixed donor and acceptor molecules placed between two electrodes. The absorption of the solar photons in the donor-acceptor mixture gives rise to electron-hole pairs which spontaneously separate into charge carriers, the latter being collected by charge-selective electrodes thereby generating electric power. Efficiencies of 14% and 15% have been recently reached [4, 5].

One of the most important and used magnitudes in OSCs is the difference between the highest occupied molecular orbital (HOMO) of the donor and the lowest unoccupied molecular orbital (LUMO) of the acceptor, which is commonly known as the HOMO-LUMO band gap, or simply gap hereafter. The difference of the ionisation potential of the isolated donor and the electron affinity of the isolated acceptor therefore gives a quite good estimation of what the gap of the compound will be, as long as both molecules interact weakly in the device. Computational high-throughput studies have been used in this context, mostly for virtual screening among different molecular candidates (see e. g. Refs. [6, 7]).

**Fig 1:**
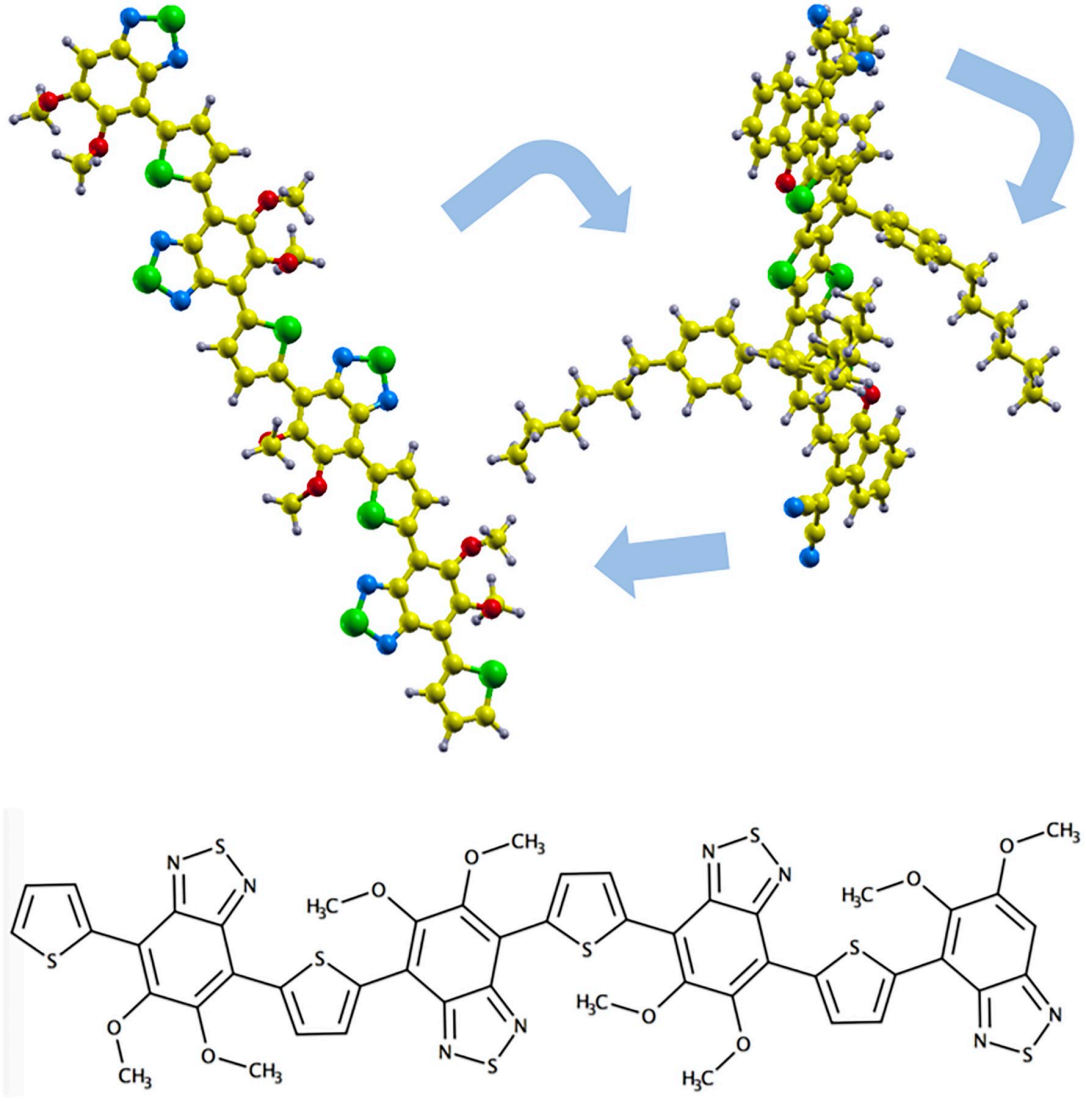
Symbolic picture of the geometry relaxation for an arrangement (up) and 4*mod* BT (down). The atoms have been represented with the following colors: C: yellow, H: grey, N: blue, O: red, and S: green.

It is well established that mutual orientation of donor and acceptor molecules at their interface in the condensed phase is not easy to predict and has paramount influence on the photovoltaic effect [8, 9]. Electronic and steric effects may hinder idealised arrangements of the isolated pairs, for instance. Predicting the packing of organic molecules in solid phases is an extremely challenging problem, well known to the pharmaceutical industry [10–12]. In this paper we do not seek to advance on the condensed-matter packing problem for organic molecules, but rather we ask the question of how does the gap change with varying packing. For the purpose, we do an ab-initio random structure search (AIRSS) [13], Fig 1 (up), by repeatedly throwing random initial orientations and arrangements of an acceptor and a donor molecule into a big enough simulation box, relaxing them, and monitoring the structure, binding energy, and gap variation of the obtained configurations.

The 4*mod* BT-4TIC complex has been considered in this study. The 4*mod* BT donor, displayed in Fig 1 (down), is composed of low-gap blocks of the polymer PBTZT-stat-BDTT-8 [14], in particular, 4 blocks of *mod* ified-benzothiadiazole (B) and thiophene (T) units. Donor-acceptors partners of this kind, formed by T-like and B-like units, have recently drawn substantial attention. Structures with tunable colors, good transmittance modulation, fast switching rate, high color efficiency, and good stability have been found [15], as well as good solubility in common organic solvents and high thermal stability [16]. Efficiencies of 4.70% and 6.60% have also been reported [17]. A very interesting finding is the ambipolar character of the TBTBT donor because of its potential applications in organic electronic devices [18]. One of the most studied acceptors is ITIC, a bulky seven-ring fused core indacenodithieno[2,3-*b*]thiophene, IT, end-capped with 3-(dicyanomethylidene)-indan-1-one, IC, and with four 4-hexylphenyl groups substituted on it [19]. 4TIC [20], which is obtained from ITIC replacing the IT core by a new six-ring fused thiophene-thieno[3,2-*b*]thiophene-thiophene, 4T, and keeping the IC and the hexylphenyl chains, offers better performance, though. Its core enhances the charge mobility and extends the absorption to the infrared region. More efficient transfer of energetic carriers and prominent structural order than ITIC have been also found. Solar cells incorporating 4TIC exhibit an efficiency of 12.62% [20] and 13.20% [21]. It is proposed to be a generator of long-lived charge species upon direct photoexcitation, as inferred from the slow ground-state bleach rise in the donor partner [22].

## Method

### Random search

The study uses a variant of the ab initio random structure search (AIRSS) technique [13], in which random mutual arrangements of the two molecules are generated and then, if all atoms are at mutual distances larger than a threshold (3 Å), the global structure is relaxed to the minimum energy in the basin of the potential energy surface (PES) it landed on. This throw-relax procedure is repeated thereby sampling PES basins describing different (meta)stable bindings between the two molecules. The structure of the separate molecules is used to define the initial configuration of any throw, allowing for random mutual disposition, including initial distance and any rotation of the separate molecules. If not discarded, the relaxation stage allows for the relaxation of all atoms.

We have considered a set of 350 arrangements of the dimer, whose initial geometries were obtained with the suite of codes Geomoltools [23] which allow the manipulation of molecule pairs, including translation, rotation, mutual positioning and mutual orientation, and which allow random throws. As ever with AIRSS there is no guarantee of having converged on the minimum energy state. The usual criterion for stopping is when the outcome does not change appreciably by further sampling. In this work, the throwing was stopped when the distribution was not showing significant variation after new trials.

### First-principles calculations

Geometry relaxations were performed using the forces on the atomic nuclei given by the density-functional-theory (DFT)[24, 25] solution of the quantum problem posed by the electrons in a Born-Oppenheimer setting. It provides an overall good description of sizable systems (in our case a total of 286 atoms; 106 of 4*mod* BT plus 180 of 4TIC) with reasonable computing times. The significant recent progress in non-local approximate DFTs including van der Waals interactions [26–31] and in their efficient implementation [32], allows ab initio predictive, systematic exploration of systems packing via relatively weak binding.

The prediction of band gaps from HOMO and LUMO energies obtained from local, semilocal, and this kind of non-local approximate exchange-correlation (XC) functionals cannot be trusted since they do not reproduce a discontinuity that should be there in the exact Kohn-Sham potential when adding an extra electron [33], which affects the difference between unoccupied and occupied single particle energies. Therefore, the absolute value of the gap is not the focus of this work. However, the changes of gap with environment, or, in particular the relative shifts of the HOMO of one molecule and the LUMO of the other are expected to be mostly affected by the change of potential in the region of each of the mentioned orbitals induced by the presence of the corresponding other molecule. These medium/long range interactions with the other partner, predominantly of electrostatic origin, are well described by the mentioned DFT XC approximations. We therefore propose using the change in gap as the one extracted from the DFT calculations themselves, at the found minimum for every independent relaxation. We have employed the Siesta DFT implementation [34] (version 4.0), which is an open-source worldwide known software, devised for efficiency.

Core electrons were replaced by norm-conserving pseudopotentials [35] which were factorized [36], the local part of each pseudopotential being optimized for smoothness [34]. DFT Kohn-Sham orbitals were expanded in a basis of numerical atomic-like functions. The particular pseudopotentials and basis sets for this work were provided by Simune Atomistics Ltd. [37], and can be found in their web site. They are reproduced in Appendix A.

The real-space integrals needed for the calculation of matrix elements of the Kohn-Sham Hamiltonian were performed using a real-space grid (or auxiliary plane-wave basis) defined by a plane-wave energy cutoff of 100 Ry. Convergence tests showed that increasing the cutoff to 300 Ry modifies the binding energy in the scale of ∼ 30 meV, and the gap within ∼ 5 meV, which are well below the scale of the corresponding values in this study. For self-consistency acceleration, the extrapolation method of Pulay [38] was used, considering six past cycles.

The Siesta code uses periodic boundary conditions, and, therefore, the pair of molecules was placed in a sufficiently large simulation box, with ample amount of vacuum around it so as to have negligible interactions among periodic images. A translation and a rotation was applied to each geometry arrangement before entering in the optimization process. The system was moved to the center of the simulation box and then rotated in such a way that the largest distance between two atoms defined the new *z* axis. In this way the volume of the box was reduced and hence the calculation time, while minimizing interactions with neighbor periodic images. The sides of the simulation box ranged between ≃34−47 Å, depending on the particular arrangement (the box was generated automatically once the initial configuration for a given arrangement was devised). Given the large box size, the k-point sampling for the periodic calculations was restricted to the Gamma point.

### Structural relaxations

A robust and efficient structural relaxation procedure is an important component of the method. The relaxation problem posed by these molecules is very ill conditioned, with coexisting very high and very low PES curvatures, the former associated to very stiff bond stretchings, the latter related to both very soft intramolecular torsions and very soft intermolecular binding. Furthermore, beyond the harmonic ill definition, some of the soft degrees of freedom can show remarkably long spans of displacement with very small variation of energy (very long, winding, narrow valleys with very small incline). This scenario represents a hard challenge to any relaxation algorithm.

A considerable effort was initially dedicated to find efficient protocols for relaxation. The final strategy adopted was the steering of each geometry relaxation through seven different steps of increasing computational expense and accuracy, including basis set, coordinate optimization algorithm, XC functional (semilocal versus non-local), and force tolerance. Speedups achieved with this protocol (as compared with the full relaxation at the highest level from the beginning) were very varied, from barely a few percent to factors of 100. The stepwise energy minimization protocol is described in Appendix B.

## Results and discussion

The values for the change in the gap, Δ*E*_*g*_, the binding energy, *E*_*b*_, and the structural data are provided in the Supporting information for the 350 obtained configurations.

### Gap and binding variation

**Fig 2:**
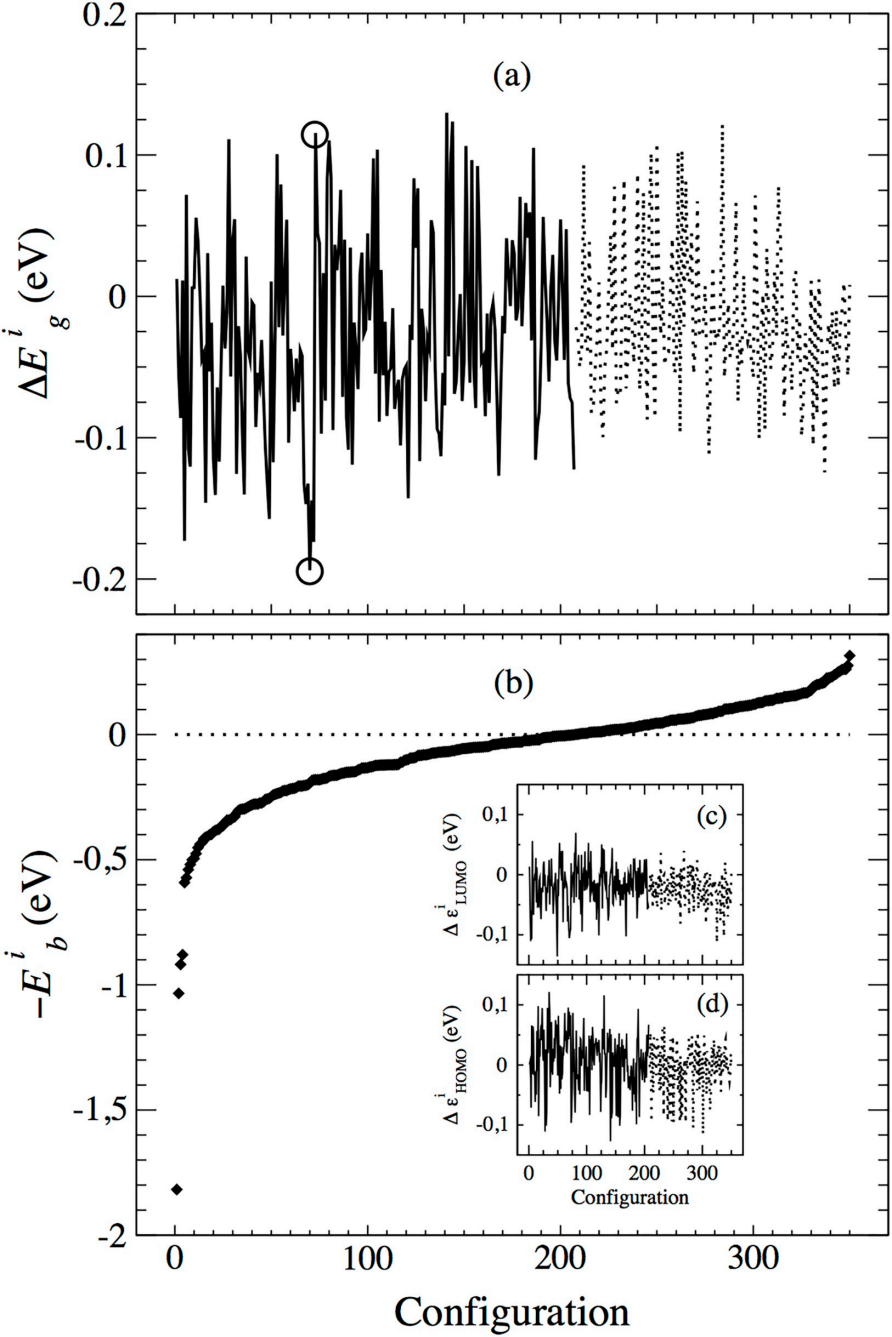
Change in the gap, Δ*E*_*g*_, (a), and binding energy, *E*_*b*_, (b), versus configuration, for the 350 arrangements considered, ordered for increasing total electronic energy. Panel (a) shows Δ*E*_*g*_ with a continuous (dotted) line for bound (unbound) pairs and the two circles single out configurations with similar binding energy but highly different gaps, for further analysis. Insets in panel (b) show the variation of the HOMO and LUMO levels, Δ*ε*_LUMO_ (c) and Δ*ε*_HOMO_ (d), respectively.

Fig 2 captures the main results of this study. We have referred both the gap and the energy to the corresponding values of the configuration in which the 4*mod* BT and the 4TIC molecules are in the same calculation box, but well separated (distance ≃18 Å). Δ*E*_*g*_ for configuration *i* is defined as

$$\begin{array}{c} \Delta E_{g}^{i}=E_{g}^{s}-E_{g}^{i}, \end{array}$$ (1)

with $E_{g}^{i}=\varepsilon_{\text{LUMO}}-\varepsilon_{\text{HOMO}}$ for the *i*−th joint configuration, and $E_{g}^{s}$ is the same for the separate dimer. Analogously, *E*_*b*_ is defined as

$$\begin{array}{c} E_{b}^{i}=E_{GS}^{s}-E_{GS}^{i}, \end{array}$$ (2)

where *E*_*GS*_ indicates ground-state total electronic energy. We adopt the usual sign convention for *E*_*b*_, with which *E*_*b*_ > 0 indicates a bound pair.

In Fig 2 both Δ*E*_*g*_ (upper panel) and −*E*_*b*_ (lower panel) are plotted for the different configurations ordered by increasing −*E*_*b*_ (or increasing *E*_*GS*_). The figure shows that there is a slight reduction of the average gap (of around 30 meV). The clearest result, however, is the much larger dispersion of this magnitude. The variability of the gap is within ±0.3 eV, an order of magnitude larger that the average shift. Furthermore, no significant correlation between Δ*E*_*g*_ and *E*_*b*_ is observed. It is therefore hard to infer *a priori* any shift of the gap based on intuition and binding. This latter finding is in agreement with previous studies on similar systems [39]. The insets in Fig 2(b) show the variation of the LUMO (panel (c)) and HOMO (panel (d)) levels, taking as reference the corresponding values for the separate dimer. The geometric mean of the widths of these two distributions amounts to ≃90% of that of the gap and suggests that the shift of the HOMO on the donor and the LUMO on the acceptor due each other’s weakly bound presence is much less affected by the DFT gap problem than the absolute value of the gap itself.

In Fig 2(b) zero binding energy is indicated with a horizontal dotted line that separates the 207 bound states on the left and the other 143 unbound states, on the right. The unbound states here defined by *E*_*b*_ < 0 are expected to be metastable since they were found as minima in the PES. The characterization of their stability would require further (and substantial) work in the evaluation of transition states out of their basins. That study is beyond the scope of the present work. We cannot even discard the possibility of some unbound states having stopped on a flatter region of the PES than detectable in the relaxation. Continuing relaxation with a substantially tighter force tolerance (one order of magnitude) for a sample of those states has resulted in no significant changes of the results, which implies that, although not discarded, unstable states among the unbound are unlikely. In any event, a more costly, tighter revision of the unbound states would not alter the results so as to affect the conclusions of this study. One could read in the figure a marginal difference between the average gap for bound states and that for unbound ones, but we think it is neither statistically significant nor reliable in the sense discussed.

The shape of Fig 2(b) is remarkable, with its lowest value at *E*_*b*_ = 1.82 eV, much deeper than the rest, which are all *E*_*b*_ ≤ 0.6 eV, except for three other configurations with *E*_*b*_ ≃ 1 eV. It should be mentioned that the *E*_*b*_ values in the figure have not been corrected for basis set superposition error (BSSE). Binding energies among molecules like ours are very sensitive to basis set convergence because of BSSE. A counterpoise correction [40] (CC) (see Appendix A) of 138 meV has been obtained for the lowest energy configuration, our prediction for the lowest binding energy becoming *E*_*b*_ = 1.75±0.07 eV (4%). The error bar accounts for the fact that the converged value is expected to be between the uncorrected and corrected binding energies.

As it is well known in AIRSS sampling, there is no way of discarding the existence of lower minima. Although our effective configurational space might appear to be of moderate dimension (given one oriented molecule, there are three Euler angles to define along what direction to find the center of the second molecule, three more Euler angles to define the orientation of that molecule, and one distance), the situation is more complicated since relative orientation couples with alteration of the original molecular shapes, especially in their flexible tails. This coupling would add a few extra soft modes to the effective number of degrees of freedom of the problem. Given the results, it is to be expected, however, that the main features of Fig 2 will remain for larger samplings. Namely, the mentioned gap variability and lack of correlation with the binding, and the funnel shape of the *E*_*b*_ curve, indicates strong but very specific binding. The particular type of binding for the lowest *E*_*b*_ is discussed in the next section.

The gap variability is due to the different contact/binding between the molecules in the distinct dispositions, including both electrostatic and hybridisation effects, or, in other words, diagonal and non-diagonal terms in the effective coupling Hamiltonian if one were to model it. The diagonal terms are longer range (algebraically decaying with distance), depending on net charges and dipole dispositions of the two molecules in each configuration. The hybridisation contributions (off-diagonal, or “hopping” terms) are much more short-range (exponentially decaying with distance), and therefore involving only the atoms of substructures of the different molecules that are very close to each other (typically under 3.5 Å). In the next section a more detailed analysis can be found for the extreme cases found in the sampling.

A question that arises is how significant is the obtained 0.3 eV gap variability. There are two sides to this question. Firstly, one could argue that if the most favorable arrangement is found, it would be irrelevant by how much that gap may change with packing in general, if we do know the gap for that particular configuration. The question is then, how likely is it to end up with the “wrong” stacking in a particular growth of the mixture of these molecules, meaning a stacking that is different from the most favorable one for the pair. The way the molecules stack is expected to be different and even variable in the condensed phase depending on crystallinity (versus disordered arrangements), and, within crystals, on possible allotropic variability. The packing/stacking/docking problem in the condensed phase is a long-standing well-known challenge in fields as important as predictive pharmaceutics. The gap variability therefore remains relevant.

Secondly, how large is 0.3 eV as compared with the value of the gap itself. The gap will depend on the particular molecules. The variability will as well, but we would conjecture that within molecules in this organic photovoltaics context it could be weakly system dependent. In our case, the gap for the 4TIC molecule is around 1.4 eV (see Appendix C), which effectively represents an upper bound to any gap with any acceptor molecule of any use for photovoltaics (with a lower LUMO energy than the one of 4TIC itself). That gives a ∼21% lower bound for the gap variability.

### Analysis of particular cases

As stated before, no clear correlation between the geometry and Δ*E*_*g*_ or *E*_*b*_ has been found. However, it is worth to analyse in detail some particular cases and explain their main features.

#### Most stable structure

**Fig 3:**
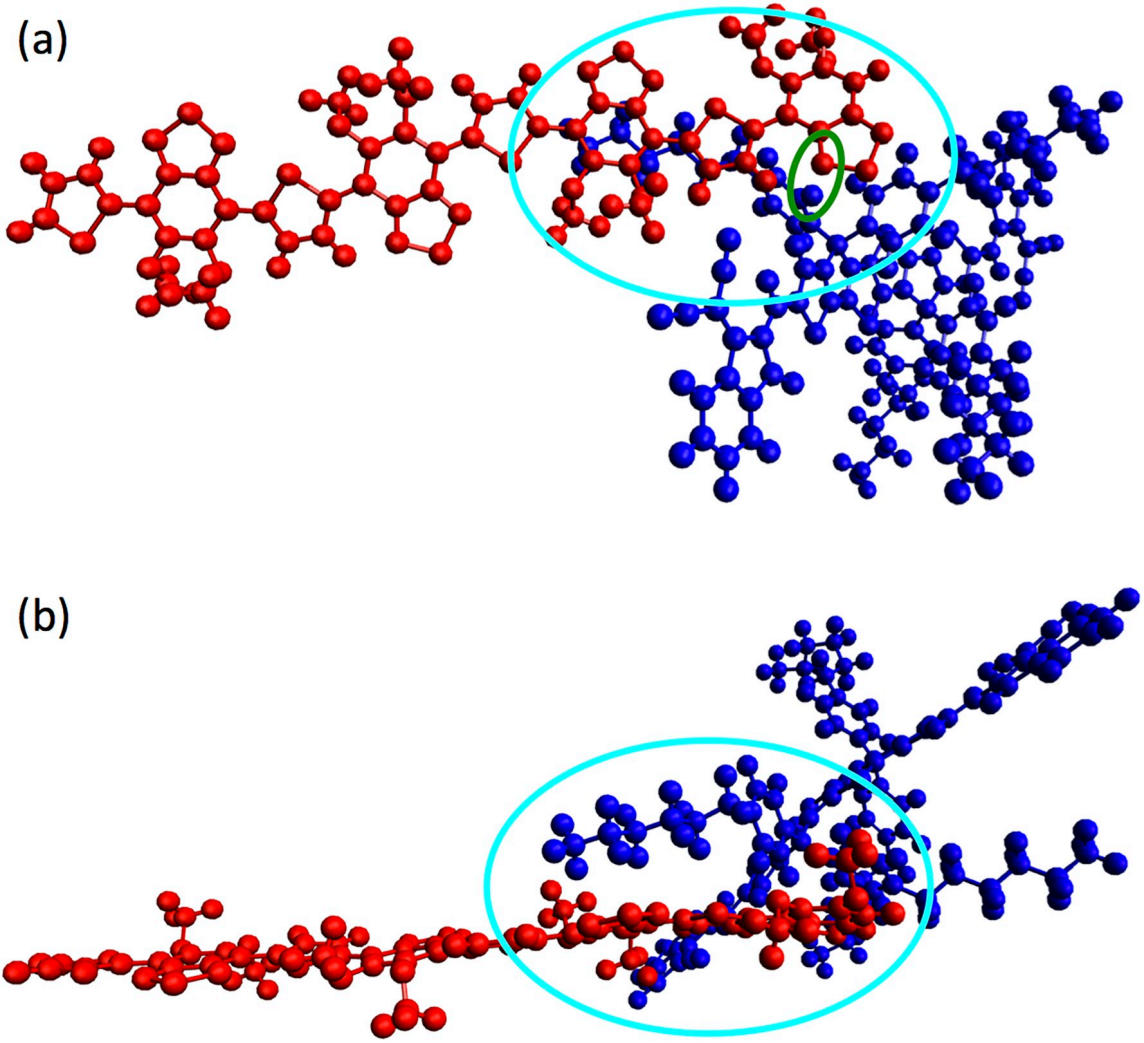
Two views, (a) and (b), for the most stable configuration of the 4 *mod* BT-4TIC dimer. Displayed in red the 4*mod* BT (donor) and in blue the 4TIC (acceptor). The overlap of the alkyl chain of the 4TIC with the aromatic core of the 4*mod* BT is marked with cyan ovals. The atoms with the shortest distance of the dimer are indicated with a green oval in (a).

The most stable arrangement is plotted in Fig 3. An overlap of one of the alkyl chains of the 4TIC with the aromatic core of the 4*mod* BT can be appreciated (cyan ovals), which gives rise to the minima distances between both molecules, involving always a hydrogen atom of the 4TIC. In particular, the minimum distance is 2.38 Å (one of the lowest found in bound cases, whose range is ∼2.2−3.5 Å) and it is the distance between a hydrogen atom of the benzene in the 4TIC and a nitrogen atom in the benzothiadiazole of the 4*mod* BT, marked in Fig 3(a) with a green oval. The second minimum distance takes place between the closest hydrogen atom to that mentioned before and the carbon atom of one of the thiophene groups of the 4*mod* BT, 2.41 Å. The third minimum distance is found between a hydrogen atom of the alkyl chain of the 4TIC and a hydrogen atom of one of the methoxy groups of the 4*mod* BT, 2.49 Å. Fig 3(b) shows the edges of the aromatic cores of both molecules, which form an angle of ∼145°, the horizontal one being 4*mod* BT. As intended, both aromatic cores are not aligned parallel to each other. This is prevented by the alkyl chain, which is also the one providing the bonding.

**Fig 4:**
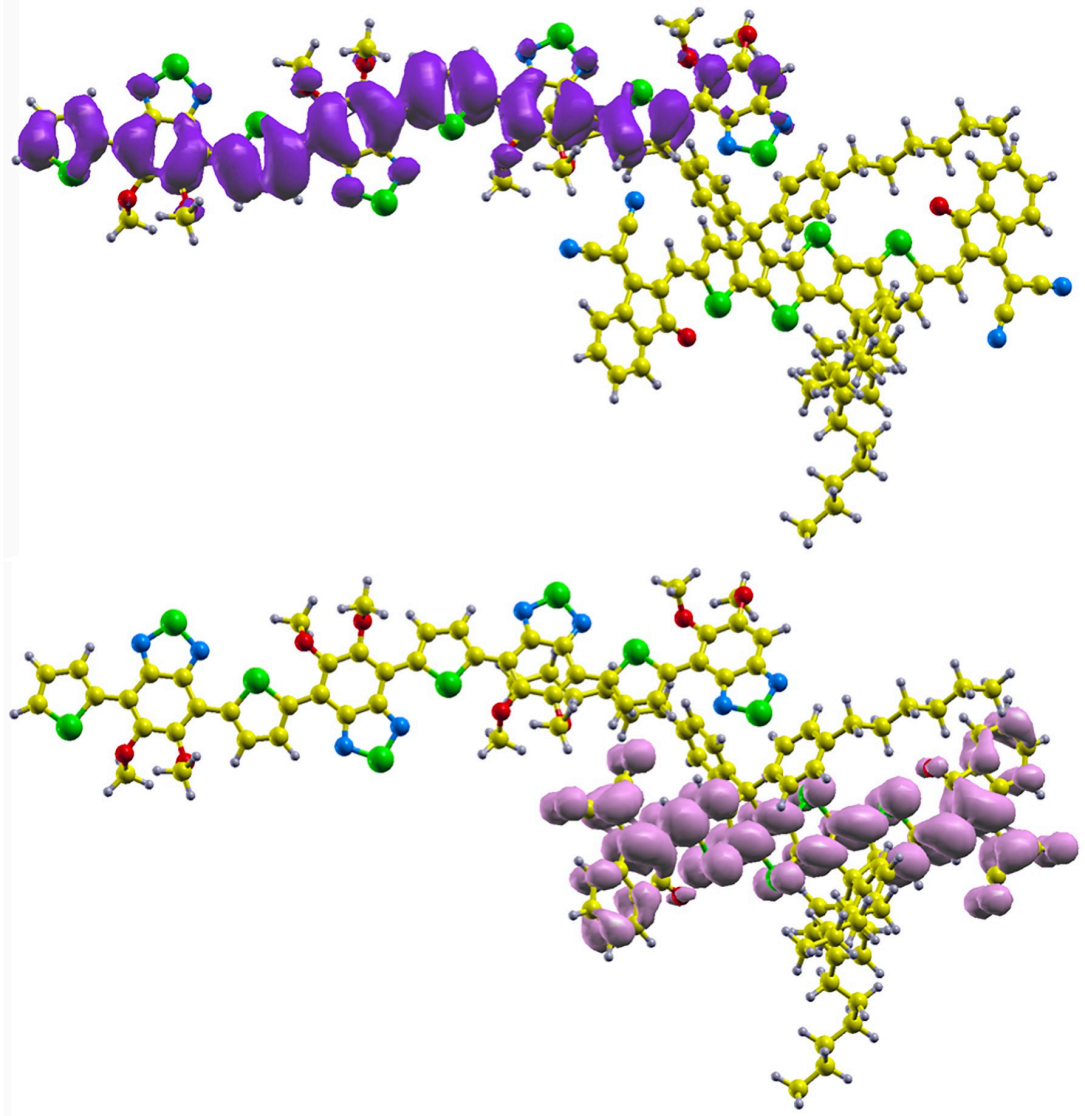
HOMO (up) and LUMO (down) levels for the most stable configuration of 4 *mod* BT-4TIC. Colors for atoms as in Fig 1.

In Fig 4, HOMO and LUMO density isosurfaces for this configuration are shown. As expected, the HOMO is located on the donor (up) and the LUMO on the acceptor (down). In the former, it is extended over the whole molecule, while in the latter only in the core. It confirms that regarding electronic effects the lateral alkyl chains of the 4TIC do not play any role, but they do from a mechanical point of view.

#### Largest gap variation

**Fig 5:**
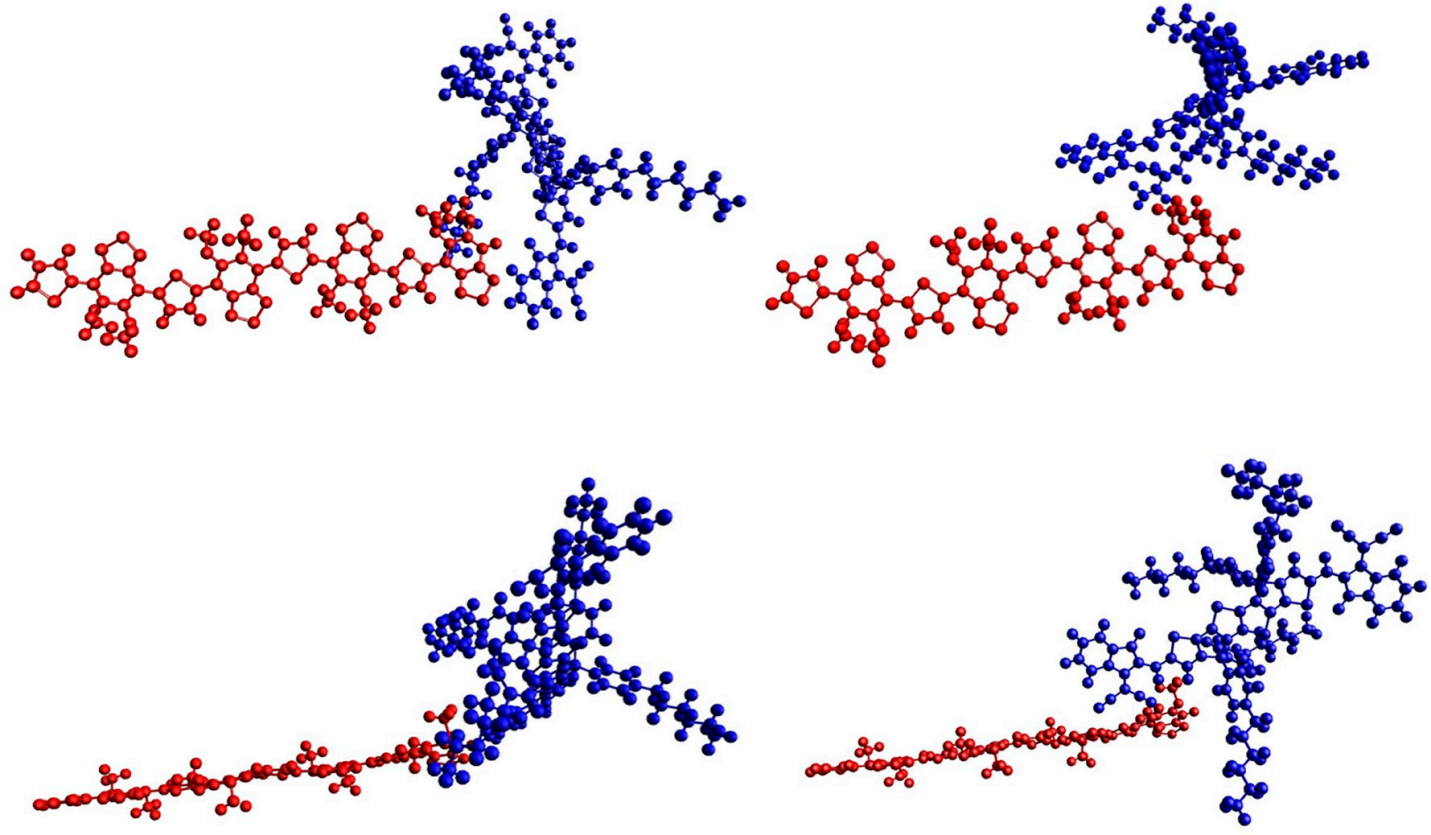
Arrangements with similar binding energy and different gap. On the left that with the gap of -0.194 eV, while on the right that with 0.116 eV. Two views are included for both configurations. Colors for atoms as in Fig 3: red for 4*mod* BT (donor) and blue for 4TIC (acceptor).

Two arrangements with very similar *E*_*b*_ but very different Δ*E*_*g*_ are displayed in Fig 5, one on the left, the other one on the right, with two different views for each (up/down). The 4*mod* BT is shown in the same position in both cases for better comparison. On the left, with *E*_*b*_ = 0.194 eV and Δ*E*_*g*_ = −0.194 eV is the configuration indicated with a circle in the lower half of Fig 2(a). On the right, with *E*_*b*_ = 0.180 eV and Δ*E*_*g*_ = 0.116 eV is the arrangement denoted with a similar circle in the upper half of the Fig 2(a). In both cases, and as in the case of the lowest energy, the aromatic cores of both molecules are neither binding nor aligning. However, the appearance of similarity of both structures is deceiving: they are quite different, although there is similarity in the relative disposition of the molecules, which is coincidental; there are all sorts of alignments in the set. In both (extreme) cases the expected situation remains, with the HOMO (LUMO) of the complex being on donor (acceptor). The one depicted on the left shows the hydrogen atoms of the indane group of the 4TIC being close to the benzothiadiazole of the 4*mod* BT, while the one on the right displays two of the methoxy groups in the 4TIC pointing to two of the benzothiadiazole groups of the 4*mod* BT.

**Fig 6:**
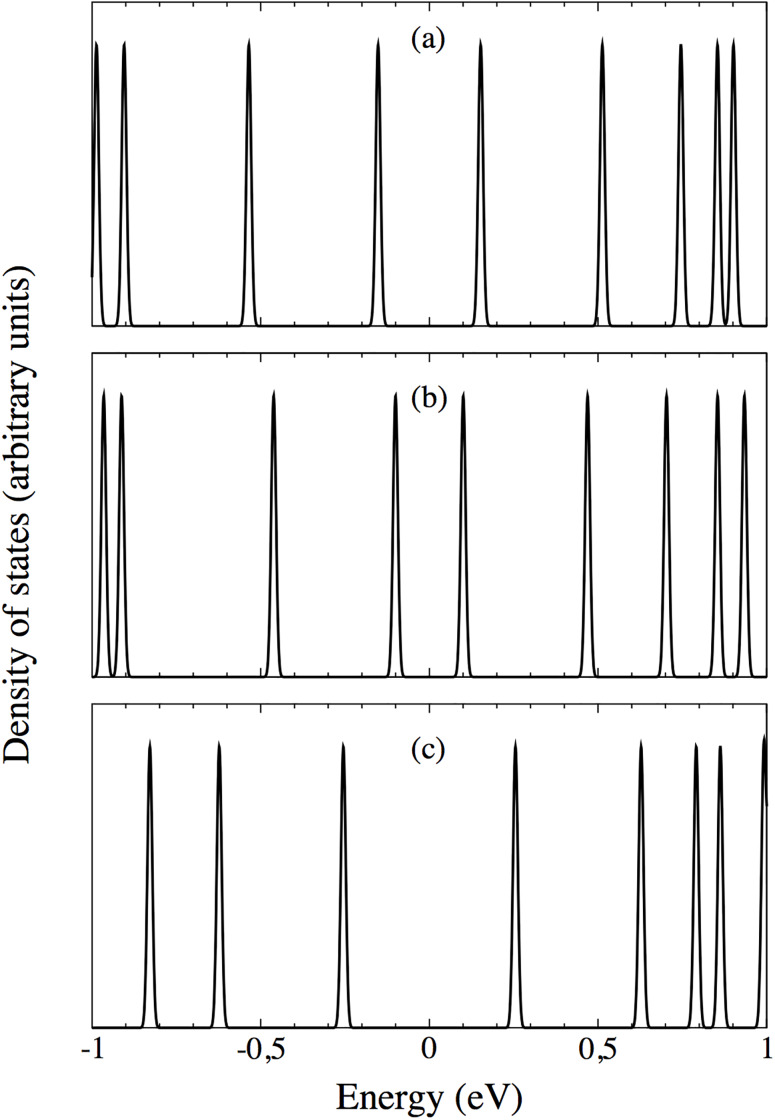
Density of states for the most stable configuration (upper panel), and for the dispositions with large gap (middle panel) and small gap (lower panel), with the energy shifted to the Fermi energy. Several levels can be seen in each plot, including *ε*_*HOMO*_ and *ε*_*LUMO*_, which are those immediately at the left and the right of the zero, respectively.

Fig 6 displays the density of states (DOS) for the two configurations analyzed in this section (middle panel: large gap, lower panel: small gap), as well as for the most stable disposition (upper panel), with the energy referred to the Fermi energy. The first peak on the left (right) corresponds to the HOMO (LUMO), and the energy difference between both gives the HOMO-LUMO gap, for these respective cases. Fig 2(a) displays their deviation with respect to the corresponding value for separated molecules, as expressed in Eq (1), to reflect the variability of such gap, rather than the actual value.

## Conclusions

In this work we have investigated the variation of the HOMO-LUMO band gap with varying packing of a 4*mod* BT-4TIC donor-acceptor molecule pair. After developing suitable tools, an ab-initio random structure search has been employed for the purpose, obtaining 350 final configurations with different initial orientations and arrangements of the donor and the acceptor species. We have arrived to the following conclusions:

1. The gap varies within 0.3 eV, which is a significant figure considering that gaps in this context are not much larger than 1 eV.
2. The gap is not found to correlate with the binding energy of the pair.
3. A clearly favoured structure with an estimated binding energy of $E_{b}^{\text{max}}=1.75\pm 0.07$ eV is found, in which an aliphatic chain of the donor aligns with part of the aromatic core of the acceptor, thereby physically separating the optically active parts of the molecules, as intended.
4. The landscape of minima shows a vast majority of weakly bonded states with $E_{b}<E_{b}^{\text{max}}/3$, with only four out of 350 with stronger binding.
5. The change of the gap, i. e. the shifts of the HOMO on the donor and the LUMO on the acceptor due to each other’s weakly bound presence, is much less affected by the DFT gap problem than the absolute value of the gap itself.

## A Pseudopotentials, basis sets, and counterpoise correction

### A.0.1 Pseudopotentials

The pseudopotentials used in this work employ the norm-conserving scheme described in Ref. [35], with the semi-local DFT form closest to the VDW-DFT used. A small non-linear partial-core correction [41] is added to the exchange-correlation computation of the overall system, to avoid near-origin oscillations. Table 1 lists the core matching radii for all species and angular momenta, and corresponding partial-core matching radii. The pseudopotentials are further factorised following the Kleinman-Bylander scheme [36], choosing a local part as described in Ref. [34].

**Table 1:** Matching radii for pseudopotentials (in Bohr).

	*r*_*s*_	*r*_*p*_	*r*_*d*_	*r*_*f*_	*r*_core_
H	1.25	1.25	1.25	1.25	-
C	1.25	1.25	1.25	1.25	1.60
N	1.25	1.25	1.25	1.25	1.37
O	1.15	1.15	1.15	1.15	1.15
S	1.70	1.70	1.70	1.70	2.60

### A.0.2 Basis sets

The basis functions used with SIESTA are products of a radial part R(r) times a spherical harmonic *Y*(*θ*, *φ*) for the angular part, which are normally (but not necessarily) centered around atomic nuclei. SIESTA accepts and numerically tabulates arbitrary radial shapes for R(r) with the only condition of their being of finite support, i. e., non-zero only within a sphere defined by a cutoff radius. The basis functions used in this work were generated as described in Refs. [34, 42, 43]. The first orbitals for valence electrons are obtained by solving the DFT problem for the isolated atom under the given pseudopotential plus a confining potential that ensures finite support, which can be hard [42] or soft [43] confinement. Double *ζ* (DZ) bases are built by adding a second basis function to each of the previously described, for the same angular momentum channels, but with a different radial shape. The ones used here were built following the method described in Ref. [42]. Table 2 presents the parameters needed to generate the basis functions used in this work.

**Table 2:** Parameters defining the basis sets used for all species. s-DZP stands for short DZP. $r_{c}^{1}$ and $r_{c}^{2}$ are the cutoff radii (in Bohr) for first and second *ζ* orbitals, respectively [42]. *V*_0_ (in Ry) and *r*_*s*_ (in percentage of $r_{c}^{1}$): parameters used for soft-confinement [43]. *V*_0_ = ∞ indicates hard confinement at $r_{c}^{1}$. *Q* and λ: parameters for additional Coulombic confining potential for polarization orbitals [44]. ‘P’: polarization orbital is obtained from a free atom in an electric field [42].

			$r_{c}^{1}$	$r_{c}^{2}$	*V*_0_	*r*_*s*_	*Q*	λ
H	DZ	1*s*	4.71	3.76	∞	-	-	-
	s-DZP	1*s*	4.71	3.76	∞	-	-	-
		2*p*	4.71	-	∞	-	P	-
	DZP	1*s*	7.39	2.56	40.0	90%	-	-
		2*p*	7.39	-	40.0	90%	9.49	1.546
C	DZ	2*s*	4.09	3.35	∞	-	-	-
		2*p*	4.87	3.48	∞	-	-	-
	s-DZP	2*s*	4.09	3.35	∞	-	-	-
		2*p*	4.87	3.48	∞	-	-	-
		3*d*	4.87	-	∞	-	P	-
	DZP	2*s*	5.95	2.51	40.0	90%	-	-
		2*p*	7.64	2.62	40.0	90%	-	-
		3*d*	7.64	-	40.0	90%	6.40	0.01
N	DZ	2*s*	3.68	2.87	∞	-	-	-
		2*p*	4.28	2.91	∞	-	-	-
	s-DZP	2*s*	3.68	2.87	∞	-	-	-
		2*p*	4.28	2.91	∞	-	-	-
		3*d*	4.28	-	∞	-	P	-
	DZP	2*s*	5.50	2.74	40.0	90%	-	-
		2*p*	6.71	2.22	40.0	90%	-	-
		3*d*	6.71	-	40.0	90%	7.51	0.07
O	DZ	2*s*	3.31	2.48	∞	-	-	-
		2*p*	3.94	2.54	∞	-	-	-
	s-DZP	2*s*	3.31	2.48	∞	-	-	-
		2*p*	3.94	2.54	∞	-	-	-
		3*d*	3.94	-	∞	-	P	-
	DZP	2*s*	5.50	2.48	40.0	90%	-	-
		2*p*	6.02	2.31	40.0	90%	-	-
		3*d*	6.02	-	40.0	90%	7.66	0.07
S	DZ	3*s*	4.17	3.50	∞	-	-	-
		3*p*	5.09	3.82	∞	-	-	-
	s-DZP	3*s*	4.17	3.50	∞	-	-	-
		3*p*	5.09	3.82	∞	-	-	-
		3*d*	5.09	-	∞	-	P	-
	DZP	3*s*	5.77	3.35	40.0	90%	-	-
		3*p*	7.41	3.59	40.0	90%	-	-
		3*d*	7.41	-	40.0	90%	2.61	0.01

DZ and s-DZP are bases of short first-*ζ* cutoff radii, which allows very efficient, but less accurate calculations. They were obtained automatically from SIESTA using default energy-shift and split-norm parameters [42], that is, 20 mRy and 0.15, defining $r_{c}^{1}$ and $r_{c}^{2}$, respectively, for all orbitals. The final DZP basis was obtained variationally [37] by minimising the total DFT energy in isolated dimers at different distances, following the protocol described in Ref. [45].

### A.0.3 Counterpoise correction

With atomic-like basis sets the well-known BSSE arises, since each molecule makes use of the basis orbitals of the other molecule to improve its own wave-functions. It gives rise to an additional lowering of the total energy when joining the molecules together, beyond the physical binding, and, therefore, gives an overestimation of *E*_*b*_. Indeed, this quantity is particularly sensitive to basis set incompleteness.

The CC [40] represents both a correction and an assessment of BSSE. It is done here for the configuration with highest binding. In its final structure, we already had calculated *E*_*b*_ from the difference of the electronic total energy of the separate molecules minus that of the joint configuration. One now calculates the energies of the separate molecules, each one in the presence of the basis orbitals of the other. The difference in energies between naked separate molecules and the separate molecules in the presence of the other’s basis gives the CC to the binding energy. The basis-set converged binding energy is normally found between the uncorrected and the corrected values.

## B Minimization protocol

The systems considered in this work represent an important challenge to energy minimization given the presence of both very high curvatures (in stiff-bond stretching) and very low ones (in weak interactions between the molecules as well as in torsions), giving rise to quite extreme ill conditioning. After extensive testing on 2,2-dimethyl-pentane (C_7_H_18_), a small molecule displaying similar characteristics, we decided for a stepwise protocol with steps of increasing levels of accuracy, for varying basis set, minimization algorithm, XC functional, and force tolerance. The sequence of steps is presented in Table 3.

**Table 3:** Stepwise geometry relaxation. The abbreviations stand for: DZ: double zeta (double-*ζ*) basis, s-DZP: short double-*ζ* plus polarization, DZP: longer DZP basis; CG: conjugate gradient relaxation; PQ: power-quench [46] dissipative molecular dynamics for relaxation; GGA: Semi-local exchange-correlation functional (XC) in the PBE generalized gradient aproximation [47]; VDW: non-local XC approximation including Van der Waals interactions [48]. *F*_*tol*_: force tolerance to stop the relaxation, in meV/Å.

Step	1^*st*^	2^*nd*^	3^*rd*^	4^*th*^	5^*th*^	6^*th*^	7^*th*^
Basis	DZ	s-DZP	DZP	DZP	DZP	DZP	DZP
Relax	PQ	PQ	CG	CG	CG	CG	CG
XC	GGA	GGA	GGA	GGA	VDW	VDW	VDW
*F*_*tol*_	38	38	38	30	30	25	20

## C Band gap of 4TIC

The 1.4 eV value for the gap quoted at the end of Section III.A is not the result of our DFT calculations, given the mentioned unreliability of DFT gaps (the band-gap problem). It has been extracted from experimental values in the literature for measurements giving the HOMO and the LUMO energy levels (from the ionization potential and from the electron affinity, respectively) of isolated monomers, both referred to the vacuum level. Table 4 summarizes the found values, which are just for 4TIC. The gap thus obtained represents effectively an upper bound for any HOMO-LUMO gap of any pair of molecules in which the HOMO is on the donor and the LUMO on the 4TIC acceptor. The 0.3 eV gap variability reported here translates into a 21% variability when considering the 1.4 eV gap value. Being the latter an upper bound, the 21% represents an effective lower bound value for the relative variability.

**Table 4:** HOMO and LUMO energy levels for 4TIC as reported in different experimental sources, and their average.

	*ε*_HOMO_ (eV)	*ε*_LUMO_ (eV)
Ref. [20]	-5.28	-3.87
Ref. [21]	-5.28	-3.87
Ref. [22]	-5.32	-3.82
Average	-5.29	-3.85

## Supporting information

S1 File Readme file of the supporting information. File explaining the Supporting information. It also contains the value of some employed magnitudes.
(PDF)

S2 File File containing the gap of the configurations. (TXT)

S3 File File containing the HOMO and LUMO levels of the configurations. (TXT)

S4 File File containing the energy of the configurations. (TXT)

S5 File File collecting all the .XV geometry files of the configurations. (TXT)
