# Supplementary material for: Gap variability upon packing in organic photovoltaics
Source: PLoS One. 2020 Jun 16;15(6):e0234115. doi: 10.1371/journal.pone.0234115 (PMC7297352; doi:10.1371/journal.pone.0234115)
Supplement: S1 File — File explaining the Supporting information. It also contains the value of some employed magnitudes. (PDF) [file pone.0234115.s001.pdf]

In the Supporting information the data related with the change in the gap, the energy, and the geometry are gathered.

In ordered-gap.txt, ordered-homo-lumo.txt, and ordered-energies.txt the values are placed by increasing electronic ground-state energy, and the corresponding configurations are indexed accordingly.

In ordered-gap.txt the last column is the change in the gap: $\Delta E^i_g = E^s_g - E^i_g$, i. e. one has to subtract to the gap of the separate-dimer the gap of configuration i, Fig. 2(a).

In ordered-homo-lumo.txt the second and third columns are the LUMO and HOMO levels, ε_{LUMO} and ε_{HOMO}, respectively, of each configuration i. The change in the LUMO/HOMO level is the energy of the LUMO/HOMO level of the separate dimer minus the LUMO/HOMO level of the configuration i, Figs. 2(c)/(d): $\Delta\varepsilon^i_{LUMO} = \varepsilon^s_{LUMO} - \varepsilon^i_{LUMO}$ and $\Delta\varepsilon^i_{HOMO} = \varepsilon^s_{HOMO} - \varepsilon^i_{HOMO}$.

In ordered-energies.txt each of those energies must be subtracted to the energy of the separate dimer to obtain the binding energy, Fig. 2(b): $E^i_b = E^s_{GS} - E^i_{GS}$.

All energies are in eV and we have employed:

E^s_g= 0.31638, ε^s_{LUMO}= –4.60676, ε^s_{HOMO}= –4.92314, and E^s_{GS}= –34399.978.

The configurations.txt file collects the 350 geometry .XV files from the Siesta calculations (without zeros). Each .XV file contains the final geometry of that arrangement, in Bohr. The labels of the atoms are: C: 1, H: 2, O: 3, S: 4, and N: 5.
